# Identifying a Muscle Activation-Matched Eccentric Intensity and Characterizing Vastus Lateralis Spectral Fatigue Using Surface Electromyography During Incremental Eccentric and Concentric Cycling: A Pilot Study

**DOI:** 10.3390/life16101695

**Published:** 2026-10-09

**Authors:** Long-Huei Lin, Kheng-Win Khor, Jong-Shyan Wang

**Affiliations:** 1 School of Physical Therapy and Graduate Institute of Rehabilitation Science, College of Medicine, Chang Gung University, Taoyuan 333, Taiwan; cosx9954022@gmail.com (L.-H.L.);; 2 Department of Physical Medicine and Rehabilitation, Heart Failure Center, Chang Gung Memorial Hospital, Keelung 204, Taiwan; 3 Research Center for Chinese Herbal Medicine, College of Human Ecology, Chang Gung University of Science and Technology, Taoyuan 333, Taiwan; 4 Healthy Aging Research Center, Graduate Institute of Rehabilitation Science, Medical College, Chang Gung University, Taoyuan 333, Taiwan

**Keywords:** muscle contraction, electromyography, exercise test, exercise tolerance

## Abstract

Eccentric cycling generates greater mechanical output with lower muscle activation and metabolic demand than concentric cycling. However, eccentric cycling intensity is commonly prescribed based on concentric cycling workloads, which may not provide comparable neuromuscular loading between cycling modes. The eccentric intensity required to match muscle activation during a clinically relevant concentric workload and its frequency-domain surface electromyographic (sEMG) characteristics remain unclear. This pilot study aimed to identify an eccentric cycling workload eliciting vastus lateralis (VL) muscle activation comparable to a submaximal concentric workload and to compare frequency-domain sEMG characteristics between cycling modes. Twenty-one healthy females completed concentric and eccentric incremental cycling exercise tests (CICET and EICET). VL sEMG was recorded throughout both tests. The workload closest to 70% of concentric peak power output (PPO) served as the reference, and the eccentric workload producing the closest normalized root mean square (RMS) amplitude was identified as the muscle activation-matched workload. RMS, median frequency (MDF), and normalized MDF slope (NMDF slope) were analyzed. A muscle activation-matched eccentric workload was successfully identified. This workload was significantly higher than the concentric reference workload (*p* < 0.001), while normalized mean RMS was comparable between conditions. EICET showed significantly higher MDF at the initial and final stages and a significantly less negative NMDF slope than CICET. Preliminary evidence suggests that muscle activation-matched eccentric cycling requires 1.69 times the power output of concentric cycling, based on the ratio of group means, while inducing less neuromuscular fatigue. Larger studies are needed to confirm these findings.

## 1. Introduction

Eccentric muscle contraction is characterized by muscle lengthening while producing force and typically occurs when the muscle resists an external load to decelerate movement [1]. Compared with concentric contraction, eccentric contraction involves distinct mechanical and neural mechanisms, enabling the generation of greater mechanical tension and force output with lower levels of muscle activation and metabolic demand [2]. Furthermore, under the same external loading conditions, eccentric contractions generally exhibit lower fatigability than concentric contractions [3]. Eccentric cycling involves resisting backward pedal rotation driven by a motor, allowing eccentric exercise to be performed safely with minimal unnecessary joint impact while enabling precise control of the exercise workload. Consequently, eccentric cycling has been widely applied in strength training, athletic performance enhancement, and rehabilitation [4].

Previous studies have demonstrated that, at the same external power output, eccentric cycling elicits lower mean and peak muscle activation of the vastus lateralis (VL) than concentric cycling [2,5,6].A Previous meta-analyses have shown no significant differences between eccentric and concentric cycling training in improving VL muscle thickness or concentric isokinetic knee extensor peak torque [7]. These findings suggest that eccentric cycling may induce training adaptations comparable to those of concentric cycling despite requiring lower levels of muscle activation, indicating greater neuromuscular efficiency. However, most of the studies included in this meta-analysis prescribed eccentric cycling training intensity based on concentric cycling exercise test results, applying the same relative-intensity approach to both eccentric and concentric cycling [4,8,9,10,11]. For example, both groups were trained at same percent of the peak power output (PPO) obtained from a concentric cycling test [4]. Because eccentric cycling typically elicits lower muscle activation than concentric cycling at the same external power output, an equivalent percentage of PPO may not represent a comparable neuromuscular load between the two cycling modes. Therefore, identifying an eccentric cycling intensity that produces a level of muscle activation comparable to that elicited by a clinically relevant concentric cycling workload would provide important evidence for the development of more appropriate eccentric cycling exercise prescriptions.

To date, no study has determined the eccentric cycling intensity or relative workload required to elicit a level of muscle activation comparable to that produced by a given concentric cycling workload. In addition, previous studies of eccentric cycling have primarily focused on time-domain surface electromyographic (sEMG) measures, such as mean and peak sEMG amplitude, whereas investigations of frequency-domain characteristics remain limited [2,12]. Further characterization of the frequency-domain responses during eccentric cycling may provide insights into the mechanisms underlying its lower fatigability compared with concentric cycling. Therefore, the purposes of this pilot study were to identify an eccentric cycling intensity that matched the VL muscle activation elicited by a reference concentric cycling workload and to examine the frequency-domain sEMG characteristics during eccentric and concentric incremental cycling exercise tests (EICET and CICET).

## 2. Materials and Methods

### 2.1. Participants

Participants were recruited from the Chang Gung University community. A total of 22 females were screened for eligibility. Participants were eligible for inclusion if they (1) were aged ≥ 18 years, (2) had a normal body mass index (BMI), and (3) had a sedentary lifestyle. Sedentary status was defined as participation in structured exercise ≤ 3 times per week and ≤30 min per session during the previous year.

**Figure 1:**
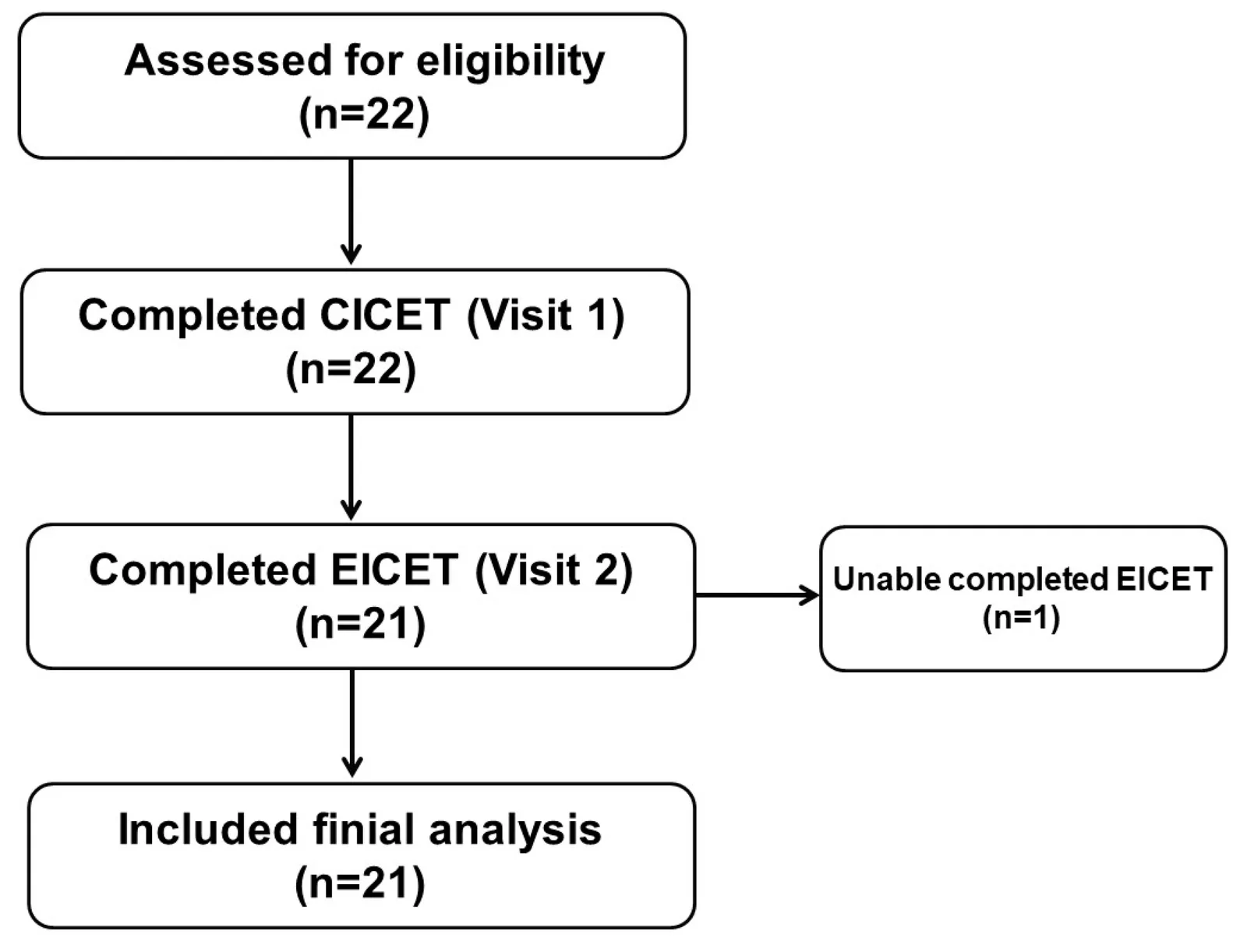
Flow diagram of participant recruitment and study completion during the concentric incremental cycling exercise test (CICET) and eccentric incremental cycling exercise test (EICET).

Participants were excluded if they (1) were considered unsuitable for exercise testing based on the Physical Activity Readiness Questionnaire for Everyone (PAR-Q+); (2) had uncontrolled cardiovascular, metabolic, neurological, or musculoskeletal conditions; (3) were taking medications that could affect muscle metabolism or contractile function; (4) were participating in another clinical trial; or (5) had any condition considered unsafe for participation. Given the pilot nature of the study, no formal a priori sample-size calculation was performed. Participants were recruited pragmatically during the predefined recruitment period, with 22 participants enrolled. One participant was unable to complete the EICET and withdrew, resulting in a final sample of 21 participants who completed both testing sessions (Figure 1).

The study protocol was approved by the Institutional Review Board of Chang Gung Medical Foundation, Taiwan (Approval No. 202600206B0), and all participants provided written informed consent before participation.

### 2.2. Study Design

This pilot study employed a within-subject repeated-measures design with a fixed testing order. All participants first completed the CICET, followed by the EICET one week later. Participants attended the laboratory on two separate occasions. During the first visit, they completed an incremental concentric cycling exercise test. Following sufficient rest after the test, participants performed a 5 min familiarization session of eccentric cycling to become accustomed to the eccentric cycle ergometer. The second visit was scheduled 1 week later, during which participants completed an incremental eccentric cycling exercise test. Before each incremental exercise test, participants performed a maximal voluntary isometric contraction (MVIC) of the knee extensors, and the highest peak torque obtained during the MVIC was used to normalize the sEMG signals. During both incremental exercise tests and the MVIC test, sEMG signals of the VL muscle were recorded.

### 2.3. Incremental Cycling Exercise Test

Participants performed both CICET and EICET (Cycling Equipment: ICE, eHuntSun Health Technology Co., Ltd., Taichung, Taiwan). Before testing, the seat height was adjusted individually to maintain approximately 5–10° of knee flexion when the pedal was at the bottom dead center. Each test began with a standardized warm-up consisting of 2 min of cycling at 30 watts while maintaining a cadence of approximately 60 revolutions per minute (rpm). Subsequently, a continuous graded exercise protocol was initiated at 30 watts. Participants were instructed to maintain a cadence of approximately 60 rpm throughout the test. The workload increased by 30 watts every 3 min until 120 watts, after which it increased by 20 watts every 3 min until the termination criteria were reached. Following test termination, the workload was reduced to 0 watt, and participants performed a 2 min cool-down. The PPO was defined as the highest workload completed for the entire 3 min stage [13]. Because the primary outcome of this study was sEMG activity, maintaining a stable pedaling cadence was essential to ensure valid comparisons across workloads. Therefore, the test was terminated if the participant was unable to maintain a cadence of 50–60 rpm for more than 5 s [14], or if the rating of perceived exertion for local leg fatigue exceeded 17 on the Borg 6–20 scale [15].

### 2.4. Maximal Voluntary Isometric Contraction Test Procedure

Maximal isometric knee extensor strength was assessed using an isokinetic dynamometer (Biodex System 4 Pro, Biodex Medical Systems Inc., Shirley, NY, USA). Participants were seated with the hip flexed to 90°, while the trunk, pelvis, and tested thigh were stabilized using straps to minimize compensatory movements [16]. The dynamometer axis of rotation was aligned with the lateral femoral epicondyle, and the knee joint was positioned at 90° of flexion for isometric knee extension testing. Participants performed three MVICs, each lasting 5 s, with 60 s of rest between contractions. The trial that produced the highest peak knee extension torque was used to normalize the sEMG signals recorded during the incremental cycling exercise tests.

### 2.5. Surface Electromyography Placement Procedure

sEMG signals were recorded using a wireless sEMG system (Biosignalsplux muscleBAN BLE, PLUX Wireless Biosignals S.A., Lisbon, Portugal), which incorporates a tri-axial accelerometer. The sEMG signals were sampled at 1000 Hz with a 16-bit resolution. Bipolar electrodes were placed over the VL according to the recommendations of the Surface Electromyography for the Non-Invasive Assessment of Muscles (SENIAM) project. Specifically, the electrodes were positioned at two-thirds of the line connecting the anterior superior iliac spine and the lateral border of the patella, with the electrode orientation aligned parallel to the muscle fiber direction. Before electrode placement, the skin was shaved if necessary, lightly abraded, and cleaned with alcohol to reduce skin impedance [17].

### 2.6. Data Analysis

**Figure 2:**
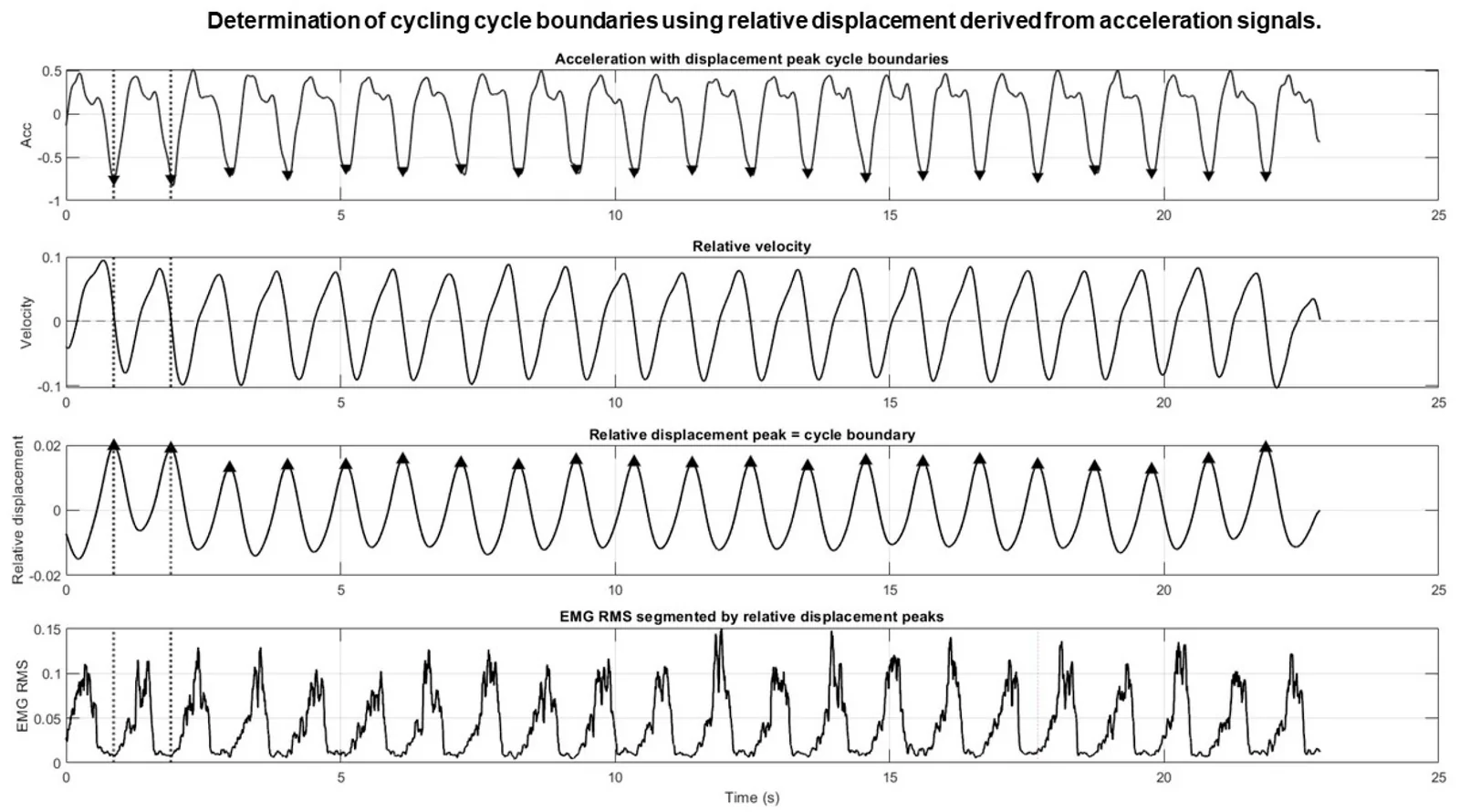
Determination of cycling cycle boundaries using relative displacement derived from acceleration signals.

During the incremental cycling exercise test, sEMG signals were recorded during the final 20–30 s of each stage. All signal processing and analyses were performed using MATLAB R2021a (MathWorks, Natick, MA, USA). An accelerometer signal was used to identify individual pedaling cycles. The acceleration signal was double integrated to obtain velocity and displacement. One complete pedaling cycle was defined as the interval between two consecutive displacement peaks corresponding to repeated velocity reversals in the same direction, thereby ensuring that each cycle represented one complete revolution (Figure 2).

The raw sEMG signals were processed using a second-order Butterworth band-pass filter (10–450 Hz) to remove low-frequency motion artifacts and high-frequency noise, followed by a 60 Hz notch filter to eliminate power-line interference. Zero-phase filtering was applied to avoid phase distortion. The filtered sEMG signals were then processed using a 50 ms moving root mean square (RMS) window to estimate muscle activation and generate a smoothed sEMG envelope [2]. For dynamic cycling analysis, the sEMG signals were time-normalized by linearly interpolating each pedaling cycle to 101 data points (0–100% of the pedaling cycle). The RMS values were subsequently normalized to the peak RMS obtained during the quadriceps MVIC. Three representative pedaling cycles were selected for analysis, and the same cycles were used for all subsequent time- and frequency-domain analyses.

For CICET, the stage with a workload closest to 70% of each participant’s PPO was selected as the reference workload to provide a standardized submaximal reference for subsequent sEMG matching with EICET. For example, if 70% PPO corresponded to 68 watts, the nearest available stage workload (60 watts) was selected. This intensity was chosen as a relatively high but submaximal workload that has been used in previous cycling studies and allows the reference workload to be individualized according to each participant’s exercise capacity. In addition, previous research has identified a reproducible VL EMG threshold at approximately 75–80% PPO during incremental cycling, beyond which VL EMG amplitude increases more steeply with increasing workload [18]. Therefore, selecting 70% PPO as the concentric reference intensity provides a relatively high workload below this reported EMG threshold, allowing muscle activation to be assessed before the steeper increase in EMG amplitude. During EICET, RMS values were calculated for each workload stage and normalized to the maximal resistance test value. The normalized RMS values obtained during EICET were then compared with the normalized RMS value at the concentric reference workload. The muscle activation-matched eccentric workload was defined as the EICET workload stage with the smallest absolute difference in normalized RMS relative to the concentric reference value. No predefined tolerance for RMS matching was applied. The within-cycle coefficient of variation (CV) was calculated from the normalized sEMG waveform across the entire pedaling cycle to quantify the variability of muscle activation within the cycle [19]. For each pedaling cycle, the standard deviation of the normalized sEMG amplitude across all 101 time-normalized points (0–100%) was divided by the corresponding mean sEMG amplitude and expressed as a dimensionless ratio.

The sEMG fatigue index was defined as the normalized median frequency slope (NMDF slope), calculated by normalizing the MDF slope to the initial MDF value [20]. The MDF slope was obtained by linear regression of MDF values across all stages of the incremental exercise test [20]. The raw sEMG signals from three trials obtained at each exercise test stage were subjected to a fast Fourier transform to determine the MDF [20,21,22]. In addition to analyzing the rate of MDF decline across the incremental cycling exercise test, MDF values at the initial and final stages were compared to determine whether differences between CICET and EICET were limited to the rate of spectral decline or were also evident in the absolute spectral characteristics at the beginning and end of the incremental cycling exercise test.

### 2.7. Statistical Analysis

All data are presented as mean ± standard deviation. Statistical analyses were performed using SPSS Statistics, version 25.0 (IBM Corp., Armonk, NY, USA), with the level of statistical significance set at *p* < 0.05. Data normality was assessed using the Shapiro–Wilk test. Normalized mean RMS, normalized peak RMS, and within-cycle CV were compared among the concentric cycling reference workload, the same-workload eccentric cycling condition, and the muscle activation-matched eccentric cycling workload. MDF values were compared between CICET and EICET at the initial and final stages of the incremental cycling exercise test. The NMDF slopes derived across the incremental cycling exercise test were also compared between incremental cycling exercise test modes. For comparisons among the three workload conditions, normally distributed variables were analyzed using one-way repeated-measures analysis of variance (ANOVA) with Bonferroni-adjusted post hoc tests. Sphericity was assessed using Mauchly’s test, and the Greenhouse–Geisser correction was applied when the assumption of sphericity was violated. Non-normally distributed variables were analyzed using Friedman tests followed by Dunn–Bonferroni pairwise post hoc comparisons. For comparisons between the two incremental cycling exercise test modes, normally distributed variables were analyzed using paired-samples t tests, whereas non-normally distributed variables were analyzed using Wilcoxon signed-rank tests.

## 3. Results

**Figure 3:**
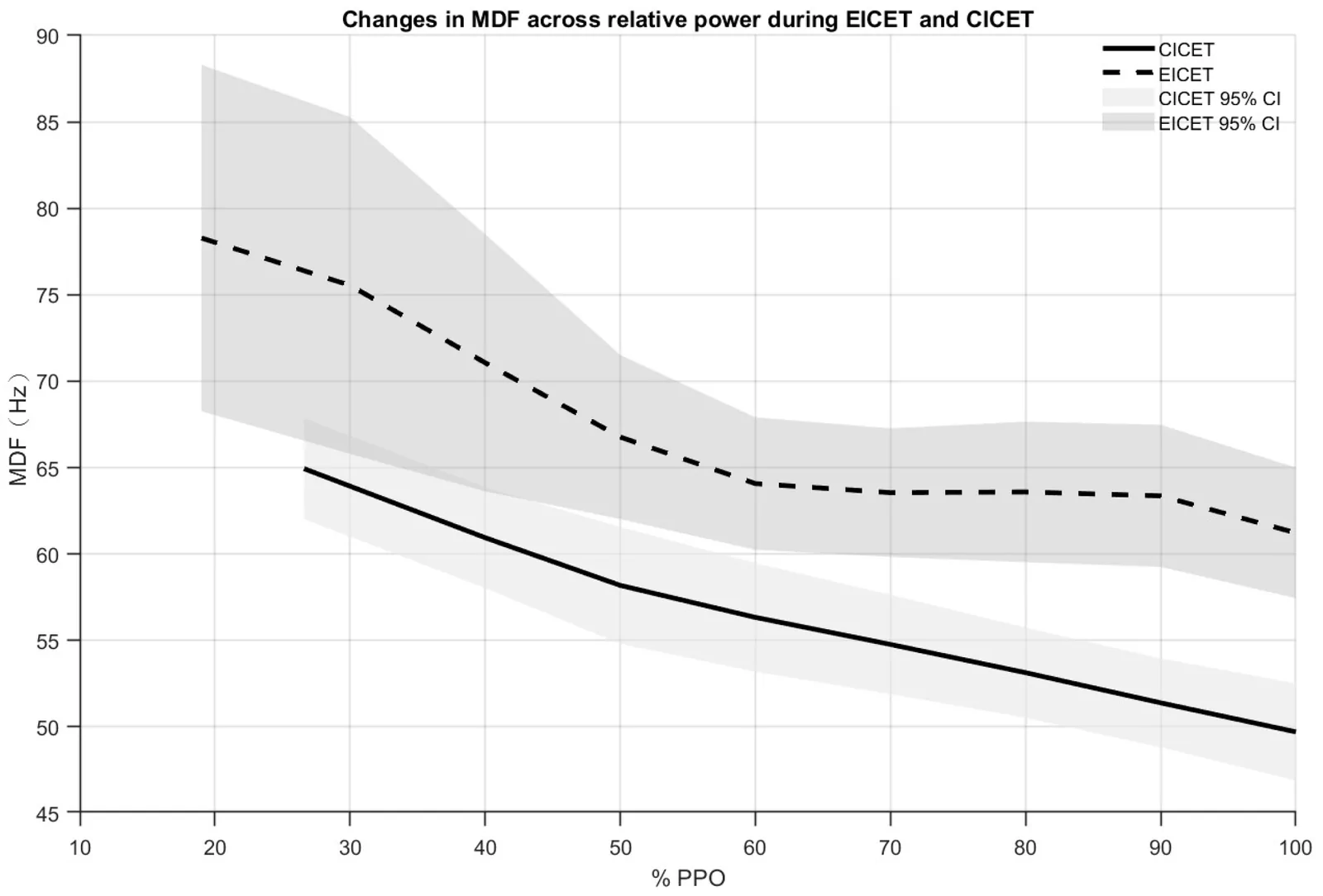
Changes in median frequency (MDF) across relative power during incremental concentric and eccentric cycling exercise tests (CICET and EICET). Power output was normalized to each participant’s peak power output (PPO). The first point of each group mean curve represents the mean MDF and mean relative workload of the individually observed initial stages, whereas subsequent MDF values were linearly interpolated at fixed 10% PPO intervals before calculating the group means. Gray lines represent individual participants, solid lines represent the group means, and shaded areas represent the 95% confidence intervals.

A total of 21 female participants were included in this study. Their mean age was 22.238 ± 2.998 years, height was 161.505 ± 5.773 cm, body weight was 55.095 ± 8.916 kg, and BMI was 21.043 ± 2.539 kg/m^2^. The results of the normality tests for all variables are presented in Table S1. Compared with CICET, EICET achieved a significantly higher PPO (160.952 ± 18.413 vs. 115.714 ± 17.911 W, Z = 4.070, *p* < 0.001). The peak MVIC sEMG amplitude used for normalization was comparable between CICET and EICET (0.267 ± 0.090 vs. 0.275 ± 0.114, Z = 0.017, *p* = 0.986). EICET exhibited significantly higher MDF than CICET at both the initial (77.819 ± 20.972 vs. 64.763 ± 6.134 Hz, Z = 2.781, *p* = 0.005) and final stages (61.301 ± 7.874 vs. 49.789 ± 5.856 Hz, Z = 3.980, *p* < 0.001). In addition, the NMDF slope was significantly less negative during EICET than during CICET (−0.001 ± 0.001 vs. −0.003 ± 0.001 stage^−1^, t = 5.358, *p* < 0.001), indicating a slower decline in MDF across incremental exercise stages (Figure 3).

**Figure 4:**
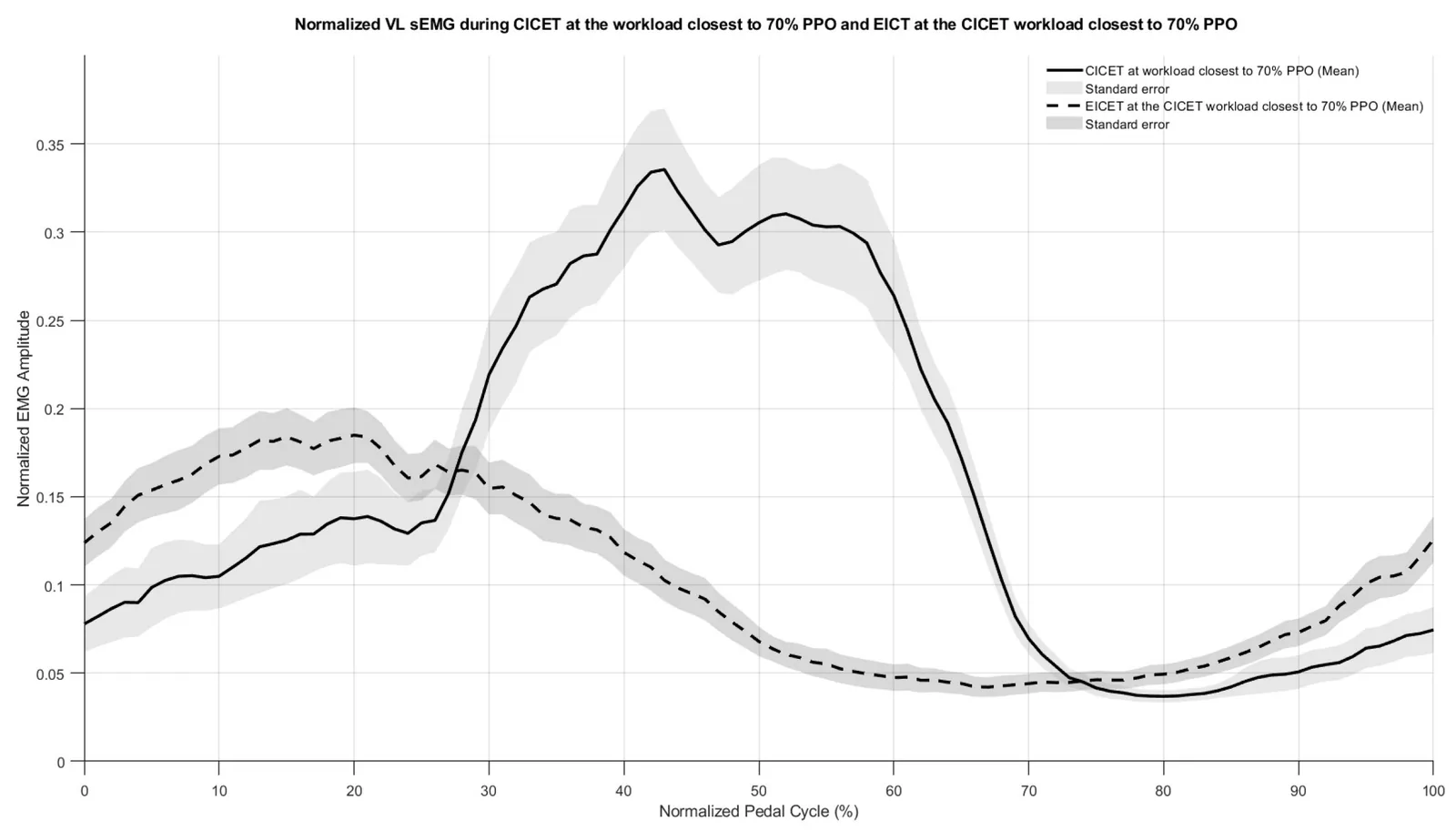
Normalized vastus lateralis (VL) surface electromyographic activity (sEMG) during the concentric incremental cycling exercise test (CICET) at the workload closest to 70% peak power output (PPO) and the eccentric incremental cycling exercise test (EICET) at the CICET workload closest to 70% PPO.

**Figure 5:**
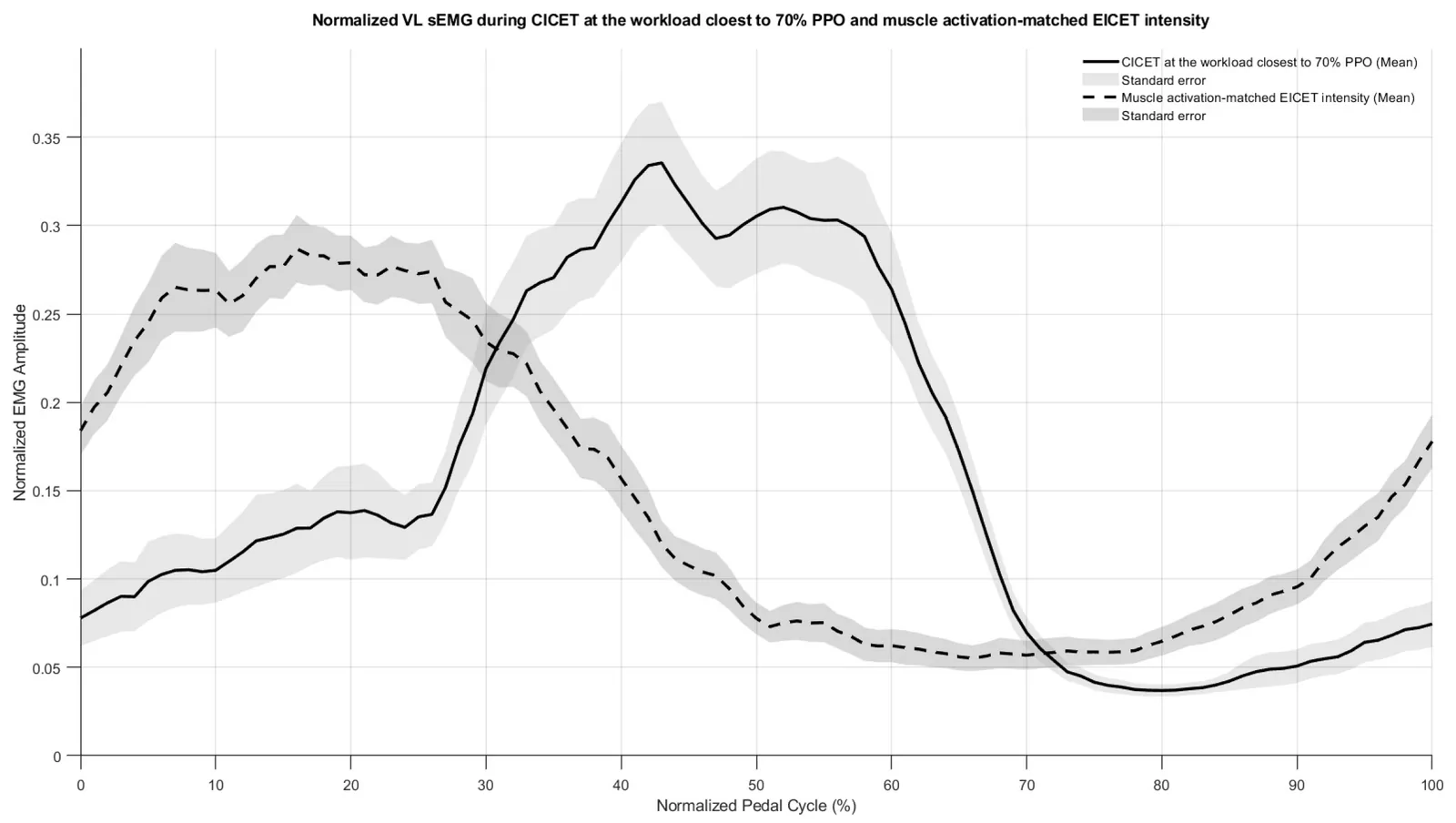
Normalized vastus lateralis (VL) surface electromyographic activity during the concentric incremental cycling exercise test (CICET) at the workload closest to 70% peak power output (PPO) and muscle activation-matched eccentric incremental cycling exercise test (EICET) intensity.

Comparisons among the CICET workload closest to 70% PPO, EICET at the CICET workload closest to 70% PPO, and the muscle activation-matched EICET intensity revealed significant between-condition differences (χ^2^ = 38.00, *p* < 0.001). The muscle activation-matched EICET intensity exhibited a significantly higher workload than both the CICET workload closest to 70% PPO and EICET at the same CICET workload (both *p* < 0.001), whereas no difference was observed between the latter two conditions (*p* = 1.000). For VL normalized mean sEMG amplitude, a significant difference was observed among the three conditions (χ^2^ = 26.632, *p* < 0.001). Post-hoc comparisons showed no significant difference between the muscle activation-matched EICET intensity and the CICET workload closest to 70% PPO (*p* = 1.000), whereas EICET at the same CICET workload showed significantly lower values than both conditions (both *p* < 0.001). Similarly, for VL normalized peak sEMG amplitude, a significant difference was observed among the three conditions (χ^2^ = 23.122, *p* < 0.001). Post-hoc comparisons showed no significant difference between the muscle activation-matched EICET intensity and the CICET workload closest to 70% PPO (*p* = 0.495), whereas EICET at the same CICET workload differed significantly from both the CICET workload closest to 70% PPO (*p* < 0.001) and the muscle activation-matched EICET intensity (*p* = 0.004) (Figure 4 and Figure 5). For within-cycle sEMG CV, significant differences were observed across all pairwise comparisons (F = 16.667, *p* < 0.001, partial η^2^ = 0.455): the muscle activation-matched EICET intensity differed from the CICET workload closest to 70% PPO (*p* = 0.027) and EICET at the same CICET workload (*p* = 0.016), while the latter two conditions also differed significantly (*p* < 0.001). Detailed descriptive statistics and pairwise comparison results for all three conditions are presented in Table 1. The effect sizes for all statistical comparisons are presented in Table S2.

## 4. Discussion

The present pilot study aimed to identify the eccentric cycling intensity required to achieve a level of VL muscle activation comparable to that observed at the concentric reference workload and to compare VL NMDF slopes between eccentric and concentric incremental cycling. Specifically, the muscle activation-matched EICET intensity was compared with the concentric reference workload, and the NMDF slopes were compared between the two cycling modes. The results demonstrated that the ratio of mean muscle activation-matched eccentric power output to mean concentric reference power output (closest to 70% PPO) was 1.695, with a significant between-mode difference. The normalized mean and peak RMS did not differ significantly between the CICET workload closest to 70% PPO and the muscle activation-matched EICET intensity, whereas the within-cycle CV showed a significant difference between the two conditions, although the *p*-value was close to the significance threshold. Regarding muscle fatigue indices, the NMDF slope during the EICET was significantly smaller than that during the incremental concentric cycling test. Furthermore, MDF values at both the initial and final stages of the incremental exercise test were significantly higher during EICET than during CICET.

To the best of our knowledge, this is the first study to determine a muscle activation -matched eccentric cycling intensity relative to concentric cycling and to compare the two contraction modes using spectral sEMG analysis. Previous studies have shown that muscle activation during eccentric cycling is substantially lower than during concentric cycling at the same mechanical workload. For example, Peñailillo et al. reported significantly lower peak and mean VL sEMG activity during eccentric than concentric cycling at comparable power outputs (*n* = 11, male) [2]. Ema found no significant difference in VL RMS-sEMG between concentric cycling at 150 W and eccentric cycling at 250 W in untrained men (*n* = 11), suggesting that approximately 1.67-fold greater power output during eccentric cycling may be required to elicit a comparable level of VL muscle activation [5]. Building on these findings, the present study identified the eccentric cycling workload required to achieve muscle activation comparable to concentric cycling and characterized distinct fatigue-related responses between the two contraction modes through spectral sEMG analysis. Furthermore, unlike previous studies conducted predominantly in men, the present study extends this investigation to women. Therefore, this study addresses an important knowledge gap in the current literature.

One possible explanation for the greater power output required during eccentric cycling to achieve sEMG activation comparable to concentric cycling is the distinct force-generating mechanism of eccentric contractions. Unlike concentric contractions, force production during eccentric contractions is not solely dependent on actin–myosin cross-bridge interactions. Instead, titin provides additional passive elastic force, thereby enhancing force production for a given level of muscle activation [23]. Previous studies have demonstrated that titin stiffness increases upon muscle activation even before force development, thereby increasing sarcomere stiffness [24,25,26]. During active muscle lengthening, force increases substantially, and the force remaining after stretch exceeds the isometric force at the same muscle length, a phenomenon known as residual force enhancement. Furthermore, the increased titin stiffness and the associated passive tension persist for several seconds after muscle deactivation, referred to as passive force enhancement [27]. Collectively, these findings indicate that titin contributes additional force beyond actin–myosin cross-bridge interactions, allowing eccentric contractions to generate greater force per unit of muscle activation [23]. Another possible explanation is the distinct neural control strategy associated with eccentric contractions. Previous studies have shown that fewer motor units are recruited and that their discharge rates are generally lower during eccentric than concentric contractions [28]. Collectively, these distinct force-generating mechanisms and unique neural control strategies may explain why a greater external workload was required to achieve sEMG activation comparable to that observed during concentric cycling.

Notably, although the eccentric workload was selected based on mean sEMG amplitude, peak sEMG amplitude was also comparable between the muscle activation-matched EICET intensity and the CICET workload closest to 70% PPO, whereas within-cycle CV differed significantly between conditions. These findings suggest that the identified eccentric intensity was comparable to the concentric workload closest to 70% PPO in terms of overall and peak sEMG amplitude, but not in the within-cycle variability of muscle activation. Therefore, although the temporal distribution of muscle activation within the pedaling cycle may remain distinct, matching based on mean sEMG amplitude may provide a practical approach for identifying an eccentric cycling intensity with comparable overall and peak sEMG amplitude, rather than an equivalent temporal pattern of muscle activation.

Another important finding of the present study was that the NMDF slope was significantly less negative during eccentric than concentric cycling. Previous studies have considered the decline in median frequency, expressed as the MDF slope, to be associated with muscle fatigue, with a less negative slope potentially reflecting a slower development of fatigue-related changes [29,30]. A possible explanation is that eccentric cycling generally imposes a lower systemic and local metabolic demand than concentric cycling at the same external power output. Previous studies have shown that oxygen consumption and heart rate are substantially lower during eccentric than concentric cycling when performed at an equivalent mechanical workload. In addition, concentric cycling at the same external workload has been reported to increase intramuscular lactate concentrations and reduce phosphocreatine stores, whereas eccentric cycling does not produce changes of a comparable magnitude [31]. Furthermore, MDF is also related to muscle fiber conduction velocity; slower conduction velocities are associated with lower frequencies in the power spectrum [32,33]. Piitulainen et al. used multi-channel sEMG to measure mean muscle fiber conduction velocity and found that it was highest during maximal voluntary eccentric contractions (4.42 ± 0.49 m/s), significantly exceeding the values observed during concentric contractions (4.25 ± 0.49 m/s, *p* < 0.01) and isometric contractions (4.14 ± 0.45 m/s, *p* < 0.001) [34]. This may partly explain why MDF was higher during EICET than CICET at both the initial and final stages.

Despite the novelty of the present study, several limitations should be acknowledged. First, pedal cycles were identified solely using an accelerometer, which did not allow precise determination of knee joint angles or examination of sEMG characteristics across different joint positions. More precise measurements of knee joint kinematics could provide additional information regarding movement coordination and the smoothness of the cycling technique. Second, only the VL was investigated in the present study. Future studies should include additional lower-limb muscles, such as the hamstrings (e.g., the biceps femoris), to provide a more comprehensive understanding of neuromuscular activation patterns during eccentric and concentric cycling. Third, the eccentric cycling data were collected after only a brief familiarization session and one full eccentric cycling bout. Although this protocol has been adopted in the aforementioned studies, it is possible that some participants had not yet achieved full proficiency in eccentric cycling, which may have influenced their muscle activation patterns. Future studies should investigate experienced eccentric cyclists to further elucidate the neuromuscular characteristics associated with proficient eccentric cycling. Fourth, only young female participants were included in this study, which limits the generalizability of the findings to males or other populations. Sex-related differences in muscle morphology, muscle fiber composition, and hormonal status [35,36] may influence muscle activation patterns and responses to eccentric and concentric cycling. Therefore, future studies should include both male and female participants to determine whether the present findings are consistent across sexes. Fifth, the relatively small sample size may have limited the statistical power to detect subtle differences, particularly for variables with greater inter-individual variability. Future studies with larger sample sizes are warranted to improve statistical power, enhance the precision of the estimated effects, and strengthen the generalizability of the findings. Sixth, the present study employed a fixed testing order, with all participants completing the CICET before the EICET. Although the two sessions were separated by one week to minimize potential carryover effects, the fixed sequence may have introduced order effects, including increased familiarity with the experimental procedures and learning-related changes in pacing or cadence control. Therefore, these potential effects cannot be completely excluded when interpreting the differences between concentric and eccentric cycling. Future studies should consider randomized or counterbalanced testing orders when experimentally feasible. Seventh, the muscle activation-matched eccentric workload was selected from the discrete workload stages included in the incremental protocol. Although matching was determined by minimizing the absolute difference in normalized RMS between eccentric and concentric cycling, no predefined tolerance for RMS matching was applied. Moreover, the relatively coarse workload increments may have limited the precision with which the muscle activation-matched intensity could be identified. Therefore, the selected workload should be interpreted as the closest available approximation of equivalent muscle activation rather than an exact physiological match. Eighth, although the same standardized electrode-placement guidelines and anatomical landmarks were used during both testing sessions, the electrodes were removed and reapplied because the CICET and EICET were performed on separate days. Therefore, small between-session differences in electrode placement and the skin–electrode interface cannot be completely excluded and may have contributed to variability in sEMG amplitude and spectral measures. Ninth, although MDF trajectories were normalized to relative power to facilitate comparisons between concentric and eccentric cycling, this approach could not fully account for differences in absolute workload, exercise duration, and the number of completed stages between the two conditions. These factors may independently influence spectral EMG responses and therefore may have contributed to the observed differences in NMDF slopes. Consequently, the differences in NMDF slopes may not be attributed solely to contraction mode. Future studies could further compare concentric and eccentric cycling under conditions matched for absolute workload, exercise duration, or the number of completed stages to more clearly distinguish the effects of contraction mode on spectral EMG responses. Finally, this study evaluated only the acute neuromuscular responses and did not investigate the long-term effects of training. Therefore, future intervention studies are needed to determine whether training at the muscle activation-matched eccentric intensity identified in the present study results in greater improvements in muscle mass, strength, and functional performance than concentric cycling.

## 5. Conclusions

In conclusion, this pilot study identified an eccentric cycling intensity that achieved comparable VL muscle activation to concentric cycling, although substantially greater mechanical power output was required. Preliminary findings also showed a less negative VL NMDF slope during EICET than during CICET, which may be associated with lower VL neuromuscular fatigue. Larger studies are warranted to confirm these findings and determine the long-term training effects of muscle activation-matched eccentric cycling. From a practical perspective, these preliminary findings suggest that, in young sedentary women, eccentric cycling intensity may need to be increased relative to concentric cycling when the training goal is to achieve a comparable level of muscle activation.

## Figures and Tables

**Table 1:** Comparison of workload and sEMG amplitude characteristics.

Comparison of Incremental Cycling Test Results				
	CICET		EICET	
PPO (watt)	115.714 ± 17.911 ^#^		160.952 ± 18.413 ^#^	
Peak MVIC sEMG amplitude used for normalization	0.267 ± 0.090		0.275 ± 0.114	
Initial MDF (Hz)	64.763 ± 6.134 ^#^		77.819 ± 20.972 ^#^	
Final MDF (Hz)	49.789 ± 5.856 ^#^		61.301 ± 7.874 ^#^	
NMDF slope (stage^−1^)	−0.003 ± 0.001 ^#^		−0.001 ± 0.001 ^#^	
Comparison of sEMG amplitude characteristics				
	CICET workload closest to 70% PPO	EICET at the CICET workload closest to 70% PPO		Muscle activation -matched EICET intensity
Incremental cycling stage watt (watts)	84.286 ± 15.694 ^b^	84.286 ± 15.694 ^c^		142.857 ± 26.931 ^bc^
VL normalized mean sEMG amplitude	0.159 ± 0.054 ^a^	0.104 ± 0.037 ^ac^		0.149 ± 0.039 ^c^
VL normalized peak sEMG amplitude	0.415 ± 0.154 ^a^	0.227 ± 0.078 ^ac^		0.348 ± 0.094 ^c^
Within-cycle sEMG CV	0.764 ± 0.115 ^ab^	0.571 ± 0.116 ^ac^		0.655 ± 0.122 ^bc^

CICET, concentric incremental cycling exercise test; CV, coefficient of variation; EICET, eccentric incremental cycling exercise test; MDF, median frequency; PPO, peak power output; sEMG, surface electromyography; VL, vastus lateralis. ^#^ Significantly different between CICET and EICET (*p* < 0.05). ^a^ Significantly different between CICET workload closest to 70% PPO and EICET at the CICET workload closest to 70% PPO (*p* < 0.05). ^b^ Significantly different between CICET workload closest to 70% PPO and muscle activation-matched EICET intensity (*p* < 0.05). ^c^ Significantly different between closest to EICET at the CICET workload closest to 70% PPO and muscle activation -matched EICET intensity (*p* < 0.05).

## Data Availability

The original data supporting the findings of this study are available from the corresponding author upon reasonable request.

## Supplementary Materials

The following supporting information can be downloaded at: https://www.mdpi.com/article/10.3390/life16101695/s1, Table S1: Shapiro–Wilk Tests for Normality of Study Outcomes, and Table S2: Effect Sizes for Comparisons Between Cycling Modes and Among Workload Conditions.

## Abbreviations

The following abbreviations are used in this manuscript:

CICET	Concentric Incremental Cycling Exercise Test
EICET	Eccentric Incremental Cycling Exercise Test
MDF	Median Frequency
RMS	Root Mean Square
sEMG	Surface Electromyography
VL	Vastus Lateralis
