# Supplementary material for: Identifying a Muscle Activation-Matched Eccentric Intensity and Characterizing Vastus Lateralis Spectral Fatigue Using Surface Electromyography During Incremental Eccentric and Concentric Cycling: A Pilot Study
Source: Life (Basel). 2026 Oct 9;16(10):1695. doi: 10.3390/life16101695 (PMC13705859; doi:10.3390/life16101695)
Supplement: Supplementary file 1 [file life-16-01695-s001.zip › life-4560150-supplementary.pdf]

Table S1. Shapiro–Wilk Tests for Normality of Study Outcomes

Outcome	Condition	*p* value
**PPO**	CICET	<0.001
	EICET	0.048
**Peak MVIC sEMG amplitude used for normalization**	CICET	0.751
	EICET	0.006
**Initial MDF (Hz)**	CICET	0.201
	EICET	0.002
**Final MDF (Hz)**	CICET	0.279
	EICET	0.010
**NMDF slope (stage^{-1})**	CICET	0.879
	EICET	0.272
**Incremental cycling stage watt**	CICET workload closest to 70% PPO	<0.001
	EICET at the CICET workload	<0.001
	Muscle activation -matched EICET intensity	0.291
**VL normalized mean sEMG amplitude**	CICET workload closest to 70% PPO	0.005
	EICET at the CICET workload	0.528
	Muscle activation -matched EICET intensity	0.004
**VL normalized peak sEMG amplitude**	CICET workload closest to 70% PPO	0.013
	EICET at the CICET workload	0.287
	Muscle activation -matched EICET intensity	0.001
**Within-cycle sEMG CV**	CICET workload closest to 70% PPO	0.912
	EICET at the CICET workload	0.375
	Muscle activation -matched EICET intensity	0.807

Table S2. Effect Sizes for Comparisons Between Cycling Modes and Among Workload Conditions

Comparison / Outcome	Effect size	Value
**CICET vs. EICET**		
Peak power output (PPO)	*r*	**0.888**
Peak MVIC sEMG amplitude	*r*	**0.004**
Initial MDF	*r*	**0.607**
Final MDF	*r*	**0.868**
NMDF slope	Paired Cohen's *d*	**1.169**
**Three-condition comparisons**		
Workload	Kendall's *W*	**0.905**
VL normalized mean sEMG amplitude	Kendall's *W*	**0.634**
VL normalized peak sEMG amplitude	Kendall's *W*	**0.551**

Note: CICET, concentric incremental cycling exercise test; EICET, eccentric incremental cycling exercise test; MDF, median frequency; PPO, peak power output; sEMG, surface eelectromyography; VL, vastus lateralis
